# Vegan diets from an allergy point of view – Position paper of the DGAKI working group on food allergy 

**DOI:** 10.5414/ALX02400E

**Published:** 2023-03-31

**Authors:** Imke Reese, Christiane Schäfer, Barbara Ballmer-Weber, Kirsten Beyer, Sabine Dölle-Bierke, Suzanne van Dullemen, Uta Jappe, Sabine Müller, Sabine Schnadt, Regina Treudler, Margitta Worm

**Affiliations:** 1 Nutrition Therapy, Munich,; 2 NutritionTherapy, Schwarzenbek, Germany,; 3 Clinic for Dermatology and Allergology, Cantonal Hospital St. Gallen, St. Gallen,; 4 Department of Dermatology, University Hospital Zürich, Switzerland,; 5 Clinic for Pediatrics with focus on Pneumology and Immunology, Charité-Universitätsmedizin – Campus Virchow-Klinikum,; 6 Allergology and Immunology, Department of Dermatology, Venereology and Allergology, Charité-Universitätsmedizin Berlin, Berlin,; 7 University Clinic Frankfurt, Clinic for Pediatrics, Frankfurt,; 8 Division of Clinical and Molecular Allergology, Research Center Borstel, German Center for Lung Research (DZL) Airway Research Center North (ARCN), Borstel,; 9 Interdisciplinary Allergy Outpatient Clinic, Department of Pneumology, University of Lübeck, Lübeck,; 10 Department of Dermatology and Venereology, University Clinic Freiburg, Faculty of Medicine, University of Freiburg, Freiburg,; 11 German Allergy and Asthma Association (DAAB), Mönchengladbach, and; 12 Department of Dermatology, Venereology and Allergology, Leipzig University Medical Center, Leipzig, Germany

**Keywords:** assessment of critical needs, allergy, vegan diet, food allergy, ultra-processed foods

## Abstract

Vegan diets are currently attracting a great deal of attention. However, avoiding animal-based foods restricts the diet and is associated with risks, the extent and medical implications of which are at present not sufficiently understood. Elimination diets represent the usual therapeutic long-term management in the presence of food allergy. In order to understand the risks of vegan diets and to discuss these critically from the perspective of food allergies, the expertise of a nutritionist/dietitian with expertise in this area is indispensable. This position paper deals with the incentives behind and the benefits of a plant-based diet. The knowledge required to cover macro- and micronutrient dietary requirements is presented. Using the avoidance of cow’s milk as an example, the challenges of adequately meeting nutritional needs are identified and (so-called) milk alternatives are evaluated from an allergy and nutritional point of view. Finally, other plant-based (substitute) products are evaluated from the same perspective, as significant protein sources in vegan diets (e.g., legumes, nuts, and seeds) are at the same time potential and potent triggers of allergic reactions. However, the allergic potential of many substitute products cannot be fully assessed at present due to gaps in research. Wheat as the most important trigger for anaphylaxis in adults is also evaluated. Finally, the increase in ultra-processed products in the (vegan) food sector and their potential consequences for the immune system are discussed.

## Introduction 

Vegan diets are more popular than ever, but avoiding all animal-based foods can carry risks. This is relevant not only for vulnerable population groups such as infants and children, who have little or no say in dietary choices, but also for people who switch to a vegan diet without being aware of the medical implications. From allergy or nutritional therapy consultations of families with children who must avoid these foods due to milk and/or egg allergy, it is known that adhering to therapeutic diets at an early age is extremely challenging. In some cases, a diet that meets nutritional requirements can only be achieved with the help of special therapeutic formulas. This is especially the case for young allergy sufferers, who reject meat and fish due to the taste or do not consume them in sufficient quantities. The same applies if not just animal-based foods but also vegetable protein sources are left out due to further food allergies. A comparable situation arises when a vegan diet is intended with concomitant allergies to nuts and/or legumes, or to birch pollen, which can result in possible cross-reactions to plant-based foods. The perspective of food allergies, in particular the expertise of nutritionists/dieticians with expertise in food allergies, regarding the consequences of elimination diets was brought together in this working group to discuss the topic of vegan diets from an allergy perspective. Many allergies to plant-based foods are persistent and may not be tolerated over a longer period of time, possibly permanently. In such cases, a vegan diet should be critically evaluated. 

## I. Advantages of a plant-based diet 

In 2008, according to the National Consumption Study II, which was conducted by the Max Rubner Institute on behalf of the then Federal Ministry of Food, Agriculture and Consumer Protection, fewer than 80,000 people in Germany (~ 1%) had completely given up animal-based products [1]. In 2022, the number had risen to 1.58 million according to a market research institute [2]. 

The decision to follow a vegan diet can have various reasons: religious traditions, health aspects, efforts to achieve sustainability, animal welfare, climate protection. In recent times, environmental and animal welfare aspects are the primary reasons for a change in diet. According to a representative survey by a market research institute in 2016, animal welfare was considered the main reason for a vegan diet in 60%. Only 8% named health aspects as their motivation [3]. Nevertheless, the health benefits of a vegetarian or even vegan diet are often highlighted making vegans believe they were safe with regard to their health. 

This is implied by data on non-communicable diseases among vegetarians or vegans: the incidence of cardiovascular disease including hypertension, type 2 diabetes mellitus, and cancer are lower than in omnivores [4]. However, these results usually do not take into account the observation that a Mediterranean diet with abundant amounts of fish, poultry, eggs, and dairy products is also associated with a significant reduction in cardiometabolic risks [5]. Modulating effects may be driven by a diet rich in nuts, vegetables, and fruits on fat and glucose metabolism [4, 6]. 

The fact that people from affluent countries, who voluntarily opt for a vegan lifestyle, are generally more health-conscious, with no or lower tobacco consumption, higher levels of exercise, and no or moderate alcohol consumption is likely to play a much more significant role than the mere avoidance of animal-derived products [7, 8]. In addition, these people have higher levels of education and income. This suggests a significant “healthy user bias”, which complicates the evaluation of prospective cohort studies regarding the significance of a vegan diet on health. 

In summary, health-conscious lifestyle factors combined with a vegetable-based diet of minimally processed foods – regardless of diet choice (vegan vs. vegetarian vs. omnivore) – lead to a health-sustaining diet [9].

**BOX 1:** The benefits of a plant-based diet may primarily be attributable to the often associated “health-conscious” lifestyle.

## II. A vegan lifestyle requires knowledge and active management 

### 1. A vegan diet requires daily consumption of legumes and nuts to meet nutrient requirements 

In a vegan diet, in order to cover the recommended daily allowance of nutrients and to prevent malnutrition, the daily consumption of legumes – especially soy – nuts, and seeds, as well as vegetables, potatoes, and whole grain products is obligatory [10]. Table 1 shows an exemplary daily eating plan for an adult. However, in view of the required quantities, this cannot be readily transferred to children. In view of this, both the German Society for Pediatrics and Adolescent Medicine (DGKJ) in its consensus paper of 2019 and the German Nutrition Society (DGE) in its position paper of 2016 urge that, in addition to the substitution of critical micronutrients and long-chain omega-3 fatty acids EPA (eicosapentaenoic acid) and DHA (docosahexaenoic acid), the intake of sufficient (high-quality) protein and energy must also be ensured [10, 11]. 

### 2. Critical micronutrients need to be supplemented in order to meet daily requirements 

Depending on its implementation, even an omnivorous diet (i.e., including animal-based foods) can be deficient in micronutrients. In a vegan diet, supplements are indispensable, even if the diet is of high quality. 

In addition to vitamin B12, which must be substituted daily, the intake of other micronutrients is critical in a purely plant-based diet. Therefore, sufficient intake of iron, zinc, iodine, calcium, selenium, riboflavin, and vitamin D, as well as the long-chain omega-3 fatty acids EPA and DHA from marine microalgae, must be ensured [10, 11]. It should be noted that on account of the poorer availability of iron, calcium, and zinc in a purely plant-based diet, the daily requirements are increased [10, 11, 14, 15]. 

Using data from the 2017 – 2018 NHANES (National Health and Nutrition Examination Survey), it was shown that with a well-planned, traditionally oriented, vegan diet, it is possible to provide most of the nutrients, with the exception of calcium and vitamin B12 [16]. However, in this work not all critical nutrients were considered: for example, no data were available on the intake of iodine and long-chain omega-3 fatty acids. 

The results of the VeChi Diet Study (Vegetarian and Vegan Children Study), a German cross-sectional study of young children aged between 1 and 3 years with different diets (vegan, vegetarian, omnivorous) show that the requirements of critical micronutrients such as calcium and iodine as well as the long-chain omega-3 fatty acids are not met in vegans and those of vitamin B2 and iron (due to poorer availability from plant sources) are only met to a limited extent [17]. The fact that the supply was also inadequate for various micronutrients in omnivorous and vegetarian-fed young children shows that there is a need for action in all children, but especially in those on vegan diets. 

A recent systematic review that examined adequate nutrient supply of vegetarian and vegan diets during complementary feeding concluded that diets that avoid or severely limit animal-based foods were not safe [18]. The available evidence suggested that the risk of deficiency of critical nutrients and growth retardation was high. There was also no evidence of any putative protective effect with regard to the development of non-communicable diseases in young children.

**BOX 2:** **Prerequisites for an adequate nutrient supply in a vegan diet. **At any age, but especially in (small) children, the prerequisites for a vegan diet that meets nutritional demands are as follows:

- Willingness to continuously supplement critical nutrients, such as vitamin B12, iodine, the long-chain omega-3 fatty acids EPA and DHA, as well as calcium, iron, zinc, and selenium, if applicable (after nutritional assessment by nutritional analysis and control of reliable laboratory parameters*)
- Uncomplicated eating behavior, no distinct likes or dislikes, good appetite
- Preferential use of high-quality, minimally processed, or natural foods
- Regular assessment of the nutritional status via nutritional analysis

*Calcium laboratory parameters are not meaningful with regard to calcium coverage.

### 3. An adequate supply of protein must be ensured 

In addition to the adequate supply of all critical micronutrients and the long-chain omega-3 fatty acids EPA and DHA, the adequate intake of protein and of all essential amino acids plays a central role if animal products are not consumed. In this respect, the experience of nutritionists/dieticians with expertise in allergies, regarding the consequence of avoiding milk and/or egg in allergic children is beneficial. In people allergic to milk and/or egg – similar to people adhering to a vegan diet – critical nutrient supply situations arise in particular, if additional aversions and/or food allergies to legumes, nuts, and/or Bet v 1-associated cross-reactivity, e.g., to soy, exist, resulting in important protein sources not being integrated in the diet. This shows that any change in diet leading to extensive avoidance – regardless of the reasons – should be supervised by allergy nutritionists/dieticians. This will allow deficits in nutrient supply to be detected early on, preventing long-term damage, and will help to develop a responsible and personalized approach to an individual nutrition in everyday life. 

**3.1 Evaluation of protein quality**

One of the critical nutrients in a vegan diet is protein, or the supply of essential amino acids (eAAs). On the one hand, an adequate protein supply results from the proportion of eAAs in plant sources which, compared to animal sources, is significantly lower. On the other hand, antinutritive substances such as protease inhibitors, phytic acid, and tannins from cereals, nuts, and legumes reduce protein digestibility. 

Since 2013, the FAO and WHO have recommended the use of the DIAAS (digestible indispensable amino acid score) as an assessment standard for protein digestibility, replacing the PDCAAS (protein digestibility corrected amino acid score) used until then [19]. In the course of the first years of life, the requirement for eAA changes, which explains the varying protein digestibility with age and illustrates the difficulty in meeting eAA needs, especially in infancy and early childhood (Table 2) [20]. The potential negative influence of thermal exposure on proteins, which is favorable in terms of inactivation of antinutritive substances, is not adequately considered with respect to protein quality [20]. 

The evaluation of protein quality is now considered more important than quantity alone, regardless of the diet. This is because increasing the quantity alone is not sufficient to adequately meet demands if the quality is lacking, especially during growth. 

Table 2 shows that legumes are better suppliers of protein compared with cereals also due to their higher protein content. Potato and soy protein show the best DIAAS values. However, the high DIAAS of potato protein should not distract from the fact that this refers to isolated potato protein, where potatoes only have a protein content of 2%.

**BOX 3:** Regardless of the type of diet – the evaluation of protein quality is more important than considering quantity alone.

**3.2 Practical implications for adequately covering protein requirements**

When high-quality animal protein is avoided – whether on account of food allergies, for ethical, and/or taste reasons – meeting protein requirements quantitatively and qualitatively is a challenge that can be addressed using four main strategies [21].

**BOX 4: Strategies for meeting protein requirements with a plant-based diet. **

Several strategies can be implemented to meet the demand for essential amino acids in vegan diets [21]:

1. Increase of consumption of a protein source, taking into account their limiting amino acids
2. Combination of protein sources with differing amino acid profiles in order to improve the amino acid profile
3. Fortification of missing amino acids to optimize the amino acid profile
4. Use of protein concentrates or isolates to reduce the amount needed to be consumed

According to **strategy 1, the consumed quantities of vegetable protein sources can** **be** **increased.** However, this often means that very large quantities need to be consumed (Table 2), which usually exceed the possible intake, especially in young children. For example, potato protein, with a DIAAS of 100, is of very high quality, but only accounts for 2% of the potato. This means that over a kilogram of potatoes would have to be consumed in order to achieve a protein serving of 20 g. 

Slightly more suitable for everyday use, but still challenging in terms of quantity, is **strategy 2, which involves combining different vegetable protein sources.** However, since the combination ratios are based on the protein content of the food (Table 2), this still means that very large quantities need to be consumed. Table 3 shows valuable protein combinations with amounts required to be consumed. However, it should be noted that the combination ratio data refer to grams of protein and not grams of food. Strategy 2 can reduce the portion size of individual protein sources but requires profound knowledge on nutrition. 

**Strategy 3, targeted supplementation of missing amino acids**. So far this has been used mainly in specialty foods and infant formulas. However, acceptance of these products may be compromised by taste. 

Currently, the **food industry is increasingly** working **with protein isolates and concentrates (strategy 4)** in order to reduce the quantities needed to be consumed or to specifically enrich products with protein. The result of this trend is that more and more highly processed foods are finding their way into vegetarian and vegan diets, and the original benefits of a diet based on vegetables, fruits, nuts, and legumes in traditional forms are being lost. This problem is discussed in more detail in the chapter “Increase in ultra-processed foods viewed critically”. 

### 4. Risk of not covering dietary requirements when avoiding cow‘s milk 

Nutritional counseling of cow’s milk-allergic infants, young children, and their families is necessary to ensure dietary requirements are met and is recommended in both national and international guidelines [22, 23]. A wide range of therapeutic formulas is available for infants, but they can only meet requirements if they are used in appropriate quantities [24]. With the introduction of complementary feeding this is not always the case but is far too rarely recognized as a problem. When introduced later (towards the end of the first year of life) therapeutic formulas are, however, often no longer accepted due to their taste or are rejected by families for other reasons. At this point at the latest, adequate coverage of daily dietary requirements, especially of calcium and high-quality protein becomes a challenge. This problem has been amplified by the frequent use of cereal and other plant-based drinks. As a result, special therapeutic formulas that are balanced and nutrient-optimized are increasingly competing with plant-based drinks that are, from a nutritional point of view, only comparable to a very limited extent. The latter are promoted with climate protection claims, some of which are controversial. Imports of these so-called milk alternatives increased from 89.5 million liters in 2017 to 206 million liters in 2020 [25]. 

In the case of cow’s milk allergy, adequate milk substitution can be achieved with the help of nutritional therapy. Allergy societies advise nutritional therapy for affected children – even beyond infancy [22]. However, if the switch from cow‘s milk to plant-based drinks is made for other reasons, e.g., a vegan diet, comparable nutritional deficits will arise. Unfortunately, awareness of this is not always present. 

**4.1 Plant-based drinks & Co – merely so-called milk alternatives**

Plant-based drinks differ, amongst other things, in the raw materials from which they are produced. Currently, retailers offer cereal drinks (e.g., made from oats, spelt, or rice), nut drinks (e.g., made from almond, cashew, hazelnut, or coconut), and drinks made from legumes (soy, lupine, pea). Drinks are also made from hemp or combinations of the above-mentioned raw materials. Mainly high-starch components from the above-mentioned raw materials are being used. For the production of plant drinks, various processing methods are used, whereby initially all raw materials undergo physical treatment. Subsequently, very different biochemical processing steps extract soluble ingredients and change macronutrient structures, among other things. For example, the starch in cereal grain is hydrolyzed by fermentation and enzymatic treatment to mono- and disaccharides, which can, depending on the final carbohydrate content, result in a sweet taste. To optimize the nutrient profile, sometimes edible oils or calcium and other micronutrients may be added. 

The range of vegetable drinks is manifold, and various additives such as flavors, vegetable oils, salt, sugar, acidity regulators, and emulsifiers are used. Depending on consumer demands regarding taste and on the technological requirements of the final product, certain additives may be added to achieve, for example, better viscosity or a product suitable for foaming. 

Preference is often given to plant-based drinks made from cereals and nuts with no added sugar. This ignores the fact that from a nutritional point of view there is no difference between sugar resulting from hydrolyzed starch and added sugar. The fact that most cereal drinks have an unexpectedly high carbohydrate content, which has an unfavorable effect on gastric retention and transit times leading to poorer nutrient availability is also ignored [26, 27]. In this respect, exclusively considering caloric intake is insufficient; caloric quality and the food matrix must also be taken into account [28].

**BOX 5: **Food matrix as well as energy origin and density impact the quality of food.

The nutrient profile of plant-based drinks differs from that of cow’s milk in terms of protein, calcium, and vitamin content so that plant-based drinks do not represent a sensible nutritional alternative to cow’s milk (Table 4) [29]. Their use in the first years of life carries a high risk for nutrient deficiencies [29, 30]. This is often not taken into account when switching from cow’s milk to plant-based drinks for sustainability reasons [31]. Therefore, when avoiding cow’s milk, allergy societies recommend preferably using a calcium-enriched soy product as a milk substitute in order to adequately meet protein and calcium requirements [22]. However, the addition of calcium to plant-based drinks is now the exception rather than the rule. In the case of soy or other vegetable-based yogurt alternatives, the practice of calcium fortification is rarer still (Table 4), so it is essential to check for an adequate supply of calcium and other valuable nutritional ingredients. 

If organic plant drinks are chosen, the risk of insufficient calcium intake is even higher. This is because, according to a ruling of the European Court of Justice, organic products must not be fortified. This includes minerals, even if supplementation would be sensible from a nutritional point of view [32]. 

For the developing market of dairy-free and vegan cheese alternatives, also nuts are used, often blanched almonds or cashews. Due to a lower content of protein and micronutrients, from a nutritional aspect, these products are not an alternative to cheese, yogurt, and cream made from cow‘s milk. Nevertheless, they may be used to meet energy requirements. The differences in terms of ingredients, processing, and nutritional content are very diverse so that an evaluation is beyond the scope of this paper. Examples of the differences – with a focus on the protein content – are shown in Table 5. 

**4.2 Soy-based milk substitutes – wrongly criticized**

Soy drinks are often rejected by parents on the grounds of alleged effects of contained phytoestrogens on sexual development [33]. This does not take into account that the feared effects have, up to the present, not been substantiated by data [33, 34] and – if at all – are only relevant as long as soy (formulas) constitute the exclusive or predominant source of nutrition. In this respect, the concerns expressed by the DGKJ are mainly limited to the first 6 months of life [35]. Soy products such as drinks, yogurt, etc. are even explicitly recommended as milk alternatives in both the *S3 guideline on allergy prevention* and the *S2k guideline on the management of IgE-mediated reactions *[22, 36].

**BOX 6: Milk substitutes. **

If the avoidance of cow’s milk is necessary or aimed at, the consumption of calcium-enriched soy drinks and calcium-enriched soy products as substitutes for cow’s milk is recommended.

**4.3 Evaluation of plant drinks with respect to allergies**

The allergic potential of plant-based “milk alternatives” depends on the plant-based ingredients used (see below). With regard to allergies, it is not always the name-giving basic ingredient that is questionable. The common practice of enriching plant drinks with isolated plant proteins to compensate for low protein content or to optimize their application is considered to be problematic from an allergy point of view. Pea protein, for example, is frequently used, which presents a particular challenge in terms of allergen labeling for people who have a pea allergy. This is because peas and products made from them are currently not subject to mandatory labeling under the Food Information Regulation [37]. 

Due to the sometimes very high degree of processing, the allergenic potential of protein isolates and concentrates cannot be fully assessed. There is a clear need for research in this area (see below). Table 6, Table 7, and Table 8 provide an overview of selected allergen structures with regard to their allergenic potential. 

## III. Evaluation of plant-based (substitute) products with respect to allergies 

With the shift away from traditional products towards meat, dairy, and egg “substitutes”, the risk for allergic and gastrointestinal reactions is also increasing. Consumers with allergies are usually able to classify traditional vegan products in terms of their composition and allergic potential and can adequately avoid individual triggers. The rapidly growing vegan market is challenging for allergy patients for a variety of reasons. Reading ingredient lists is essential, now more than ever, even though many forms of meat, dairy, and egg “substitutes” contain ingredients that were not previously classified as being of allergenic potential. 

To date, only some pulses and seeds are explicitly required to be labeled as allergen sources. Another pitfall for allergic consumers arises from the voluntary labeling of unintentional allergen cross-contact. This is particularly relevant for families who specifically select vegan products due to an allergy to animal-based foods in order to avoid that specific allergen (see below). 

It should also be noted here that the allergenic relevance of many vegan “substitute products” cannot always be fully assessed due to the, at times, very high degrees of processing. There is a clear need for research in this area (see below). The subsequent evaluation of various plant-based foods and products made from them should be seen as an attempt to estimate their allergic potential. 

### 1. The label “vegan” does not mean “free from…” 

Labeling a food as “vegan” is a voluntary food information. The Regulation (EU) 1169/2011 on the provision of food information to consumers states in Article 36 that criteria shall be established to define this [37]. Until this definition is established, the general conditions are that the voluntary information must not be ambiguous or misleading and should be based on scientific data. Vegan foods are characterized by the absence of animal-based ingredients. Therefore, patients with allergies to animal-based allergens such as milk or egg assume that foods labeled “vegan” are safe for them. However, the label “vegan” only refers to the absence of foods of animal origin as ingredients, i.e., the deliberate use of animal-based products. Following an agreement between the German State Ministries for Food and Consumer Protection, the Food Inspection Authorities, and Proveg/European Vegetarian Union, the V-Label, Germany‘s most widespread vegan label, does not take unintended, process-related cross-contact into account. 

“The inclusion of Precautionary Allergen Labelling (PAL) on the packaging (of vegan food) does not fundamentally contradict the use of the V-label. However, it is recommended to design the production in a way that a declaration is not necessary.” (Quote from the information sheet on inspections to check compliance with the V-Label guidelines https://www.v-label.eu/de/unternehmen/v-label-inspektion). 

In a study of 30 foods labeled as vegan, the DAAB found that in particular products from the confectionery/chocolate/bar sector unintentionally contain amounts of milk that can lead to allergic reactions in people with milk allergy [38]. 

Therefore, vegan labeled foods cannot be recommended to patients who react to animal allergens. Since many (allergic) consumers assume that vegan foods are free from animal-derived products, appropriate education is necessary. Especially consumers with milk allergy should be made aware of the high-risk products such as dark chocolate and vegan foods. 

### 2. Anaphylaxis to plant-based foods 

**Figure 1:**
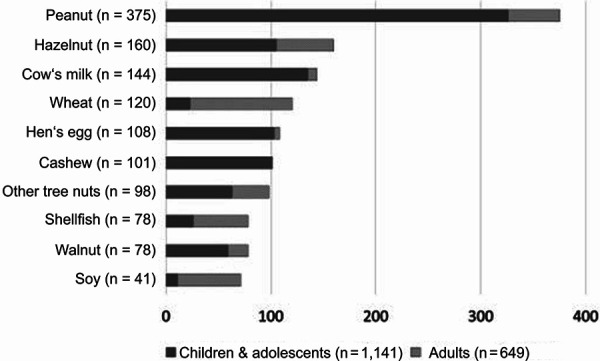
10 most frequent triggers of food-induced anaphylaxis. Anaphylaxis Registry, German-speaking countries (Germany, Austria, Switzerland), as of October 29, 2020. Total n = 1,790 confirmed food-induced anaphylaxis cases (n = 1,141, children and adolescents 0 – 17 years; n = 649, adults 18 years and older). Only cases that met the NIAID/FAAN (National Institute of Allergy and Infectious Diseases/Food Allergy and Anaphylaxis Network) definition [40] of an anaphylactic reaction and where the trigger was diagnosed as confirmed [39] were included.

While in childhood, both animal- and plant-based foods such as cow’s milk, hen’s egg, peanut, hazelnut, and cashew are common causes of anaphylaxis, in adults, plant-based foods such as wheat, hazelnut, soy, and celery predominate as triggers (Figure 1) [39]. 

**Figure 2:**
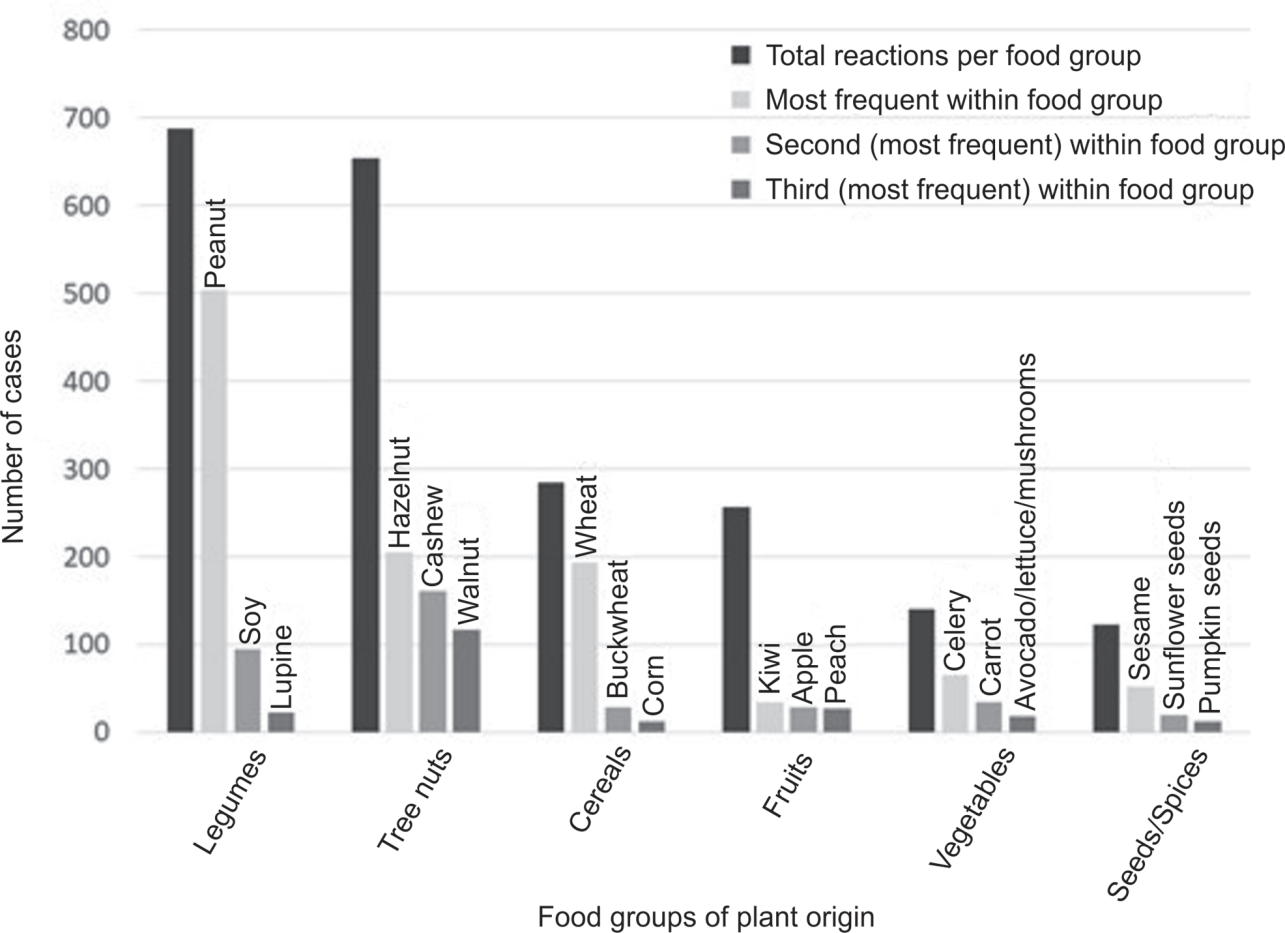
The most frequent triggers or groups of triggers in confirmed anaphylaxis to foods of plant origin. Based on [41].

Thus, with increasing age, plant-based foods are a significant cause of anaphylaxis, with the most frequent triggers being found precisely in those two food groups from which the protein supply is derived in a vegan diet, namely legumes and nuts (Figure 2). As there is great variety within the vegetable and fruit food groups, suitable alternatives can readily be found in both groups when required. Allergies to legumes, nuts, but also seeds and wheat not only restrict the menu and the quality of life, but also the nutrient supply to a much greater extent. Therefore, these groups will be discussed in more detail below. 

### 3. Evaluation of legumes with regard to allergies 

Legumes are an important source of protein in the vegan diet as well as in many different regions of the world. At the same time, they are known to provoke IgE-mediated allergic reactions, especially peanut, followed by soy [22, 39, 42]. The spectrum of allergenic legumes varies with geographical location and food consumption habits. For example, lentil occurs more frequently in Mediterranean countries and chickpea in India as the main allergen source among legumes [43]. Cross-reactions amongst legumes are recognized but of less clinical relevance than suspected. In a retrospectively analyzed cohort of peanut-allergic children, the frequency of a clinically relevant cross-reaction to other legumes was only 7.9% (order of frequency: pea, lentil, soy, sweet lupine, chickpea; no cross-reaction to bean), despite serological cross-sensitization being present in more than 60% [44]. 

**3.1. Peanut**

Peanut (*Arachis hypogaea*) belongs to the legume family. As described in the chapter on peanut allergy of the Molecular Allergology User’s Guide (MAUG) 2.0 [45], the main areas of cultivation for peanuts are China, India, and the USA. In Europe and the USA, peanuts are consumed almost exclusively roasted. They can be consumed whole or as peanut butter, peanut flips, or as an ingredient in various products. Peanuts are either roasted in the shell or alternatively shelled, blanched, and then further processed either dry or roasted in oil. To make peanut flips, corn rolls are sprayed with a paste of roasted peanuts. In other parts of the world, such as Asia and Africa, raw peanuts are used as a cooking ingredient. 

Peanut is a common cause of food allergy. Peanut allergy can occur in infancy, with existing atopic dermatitis being an important risk factor [46, 47]. Because natural development of tolerance is rare and remission occurs in only ~ 20% [48], allergic reactions can occur at any age. These involve multiple organ systems in 70% of pediatric patients [49]. Anaphylactic reactions have been described at all ages [50]. In children and adolescents, peanuts together with tree nuts account for one third of fatal reactions and in adults for half [51]. 

To date, 17 peanut allergens have been registered in the allergen database of the WHO/IUIS Allergen Nomenclature Subcommittee, with the seed storage proteins Ara h 1, Ara h 2, and Ara h 3 appearing to be the most important allergens. In addition to peanut extract-specific IgE, the determination of Ara h 2-specific IgE has proven useful in routine diagnostics [52, 53]. 

According to the Food Information Regulation (EU) 1169/2011, peanuts must be declared as an allergen source in the EU, so they and all products made from them must be labeled if they are used as an ingredient, i.e., if they are deliberately part of the recipe. Nevertheless, the widespread use of peanut in the food industry poses a risk for the consumption of allergen due to unintentional cross-contact or “hidden” peanut, and accidental allergic reactions are common [54]. 

**3.2 Soy**

Soybean (*Glycine maxima*) belongs to the legume family and is now grown on 6% of the global agricultural land. It is the world’s most important legume, impressing with a versatile nutritional profile [12]. 

The data of the German-language anaphylaxis registry (Figure 1) depict a rather low relevance of soy allergy in childhood of 1% [22]. While the clinical relevance of cross-reactions among storage proteins is limited, cross-reactions between Bet v 1 and Gly m 4 are observed much more frequently [55, 56]. Although the symptoms of pollen food syndrome to soy are usually limited to local symptoms such as oral contact urticaria, severe anaphylactic reactions can occur with rapid intake of large amounts of protein [56]. Soy is also one of the allergen sources subject to declaration according to Food Information Regulation (EU) 1169/2011. 

In the allergen database of the WHO/IUIS Allergen Nomenclature Subcommittee, various allergens from several allergen families are now registered by name, with Gly m 1 (hydrophobic storage protein), Gly m 2 (defensin), Gly m 3 (profilin), Gly m 4 (PR-10 protein), Gly m 5 (7S vicilin), Gly m 6 (11S globulin), and Gly m 8 (2S albumin) being highlighted (Table 7). Also of clinical importance is the existing cross-reactivity between Gly m 4 and the main birch pollen allergen Bet v 1 as well as between Gly m 5 and Gly m 6 and other 7S vicilins or 11S globulins (for example Ara h 1 or Ara h 2 of peanut). 

Soy and products made from soy can be processed in a variety of ways as they meet different technological requirements. As a low-cost protein source, soy ingredients can be found in many processed foods such as meat and meat substitutes, baked goods, or breakfast cereals [57]. 

In dried soybeans – depending on the variety – a protein content of 35 – 37 g/100 g can be assumed. A soy drink contains 4 g protein/100 mL (Table 2, Table 4, and Table 6). Protein concentrates and isolates contain significantly higher amounts. The concentration of Gly m 4 increases with ripening and storage of soybeans [58]. The process of heating can reduce the Gly m 4 content, but not the content of storage proteins. However, as procedures are highly variable, the risk of relevance for PR-10-allergic individuals should be considered despite processing. Products in which the protein fraction of soy is separated (for example, refined soybean oil, soy lecithin) do not pose a risk to soy-allergic individuals [57]. 

In double-blind, placebo-controlled oral food challenges (DBPCFC) of 56 Bet v 1-sensitized patients in the BASALIT (Birch Associated Soy Allergy and Immuno-Therapy) study, objective symptoms occurred in 79% of participants and subjective symptoms in 91% [59]. The triggering dose for objective symptoms ranged from 0.7 to 9.7 g of soy protein in 33 of 56 participants, and from 0.7 to 2.2 g for subjective symptoms in 29 participants. The average Gly m 4 content of the food challenge was 178 µg/g [60]. 

**3.3. Lupine**

Lupine belongs to the legumes and is phylogenetically related to peanut, soybean, pea, bean, fenugreek, etc.. Lupine seeds for consumption are obtained mainly from three different species: *Lupinus albus* (white lupine), *Lupinus angustifolius* (blue lupine), and *Lupinus luteus* (yellow lupine). Lupine flour has been consumed in several EU countries since the 1990s, both as a food and as an additive to other flours and foods. 

The first case of an immediate-type allergy to lupine in a peanut-sensitized patient was reported in the USA in 1994 [61]. In 2007, lupine allergy was described for the first time in Germany [62]. This patient was not sensitized to peanut. In a study of 39 peanut-sensitized patients, 31 were also sensitized to lupine [63]. Clinical relevance was demonstrated in 9 patients by DBPCFC with lupine flour, with the lowest triggering dose being 0.5 mg. Since 2006, lupine and products made from it have been subject to mandatory declaration. Like soy and peanut, it is included in Annex II of the Food Information Regulation (EU) 1169/2011 in the list of substances that cause allergies and intolerances and require labeling. 

To date, only three individual allergens from three protein families have been documented by the WHO/IUIS Allergen Nomenclature Subcommittee (www.allergen.org): Lup an 1 (beta-conglutin), and more recently Lup an 3 (lipid transfer protein) and Lup a 5 (profilin) [64]. Currently, only the total extract of *Lupinus albus* (ImmunoCAP, ThermoFisher Scientific (Uppsala, Sweden) can be used for routine diagnostics; individual allergens are not yet available. For prick testing, a suspension of lupine flour is used (prick-to-prick testing), as there are no approved commercially available skin test solutions. 

Despite the fact that there are severe allergies to lupine, foods containing lupine are on the rise. Widespread use carries the risk of unintentional ingestion of lupine allergens in the sense of “hidden allergens”. This is especially true for possible cross-reactions in peanut-allergic patients, who are not informed about this potential. De novo sensitizations to lupine have been described with almost comparable frequency to cross-reactions with peanut [65]. A very recent study investigated the extent of cross-reactivity among legumes in 195 peanut-allergic children with the result that sensitization to lupine, fenugreek, soybean, and lentil was found in descending order of frequency. For 27.9% of the children, at least one additional true legume allergy could be diagnosed, with the responsible triggers being lentil, lupine, or pea [66]. 

**3.4. Pea**

Pea is increasingly being used by the food industry for human nutrition and is promoted as a vegan, and in some cases hypoallergenic, source of protein, as peas are not one of the 14 major triggers of allergies and intolerances that require declaration. 

Pea is usually consumed in cooked form. Unlike peanut, the cooking process does not seem to affect the allergenicity of the major allergens Pis s 1 and 2 [67]. Other studies suggest that the temperature increase of the cooking process is already sufficient to increase allergenicity in pea, due to changes in protein structure resulting in the formation of new allergens [68]. 

Seven allergens have been described for pea (*Pisum sativum*) (Table 7), three of which are officially recognized allergens (Pis s 1, Pis s 2, and Pis s 3; www.allergen.org). To date, no single allergen is available for in vitro diagnosis. The storage proteins Pis s 1 and 2 are thought to be the major pea allergens [69]. Pis s 1 has been confirmed as the major allergen in a small, very well-characterized cohort of children [70]. Unlike other 2S albumins (e.g., Ara h 2 in peanut, Ana o 3 in cashew), the one identified in pea may not represent a relevant allergen [70, 71]. 

Interestingly, a Bet v 1-homologous allergen has also been described. Depending on the food processing procedure, the allergen may be absorbed intact, similar to Gly m 4 of soy [56], especially when consumed in protein drinks which may be the cause of anaphylactic reactions to pea protein. 

Due to its technological functional properties, pea is currently a preferred raw material for use in vegetarian and vegan products. 

**3.5 Fenugreek**

Fenugreek (*Trigonella foenum-graecum* in the *Fabaceae* family) belongs to the legumes. The dried seeds are roasted and used whole or as flour, as food or in traditional medicine. 

As a food, fenugreek is consumed in the form of spices (e.g., in curry), cheese, baked goods, confectionery, and also in coffee substitutes and herbal teas [72]. In 1997, severe reactions after ingestion, inhalation, and external application of fenugreek seed powder were published for the first time [73]. Meanwhile, anaphylaxis to fenugreek in curry spices has also been published [74, 75]. 

The allergens have not yet been characterized in detail. Faeste et al. [72] found major allergens in fenugreek with molecular weights of 50, 52, and 74 kDa. In a follow-up study, the 50-kDa protein was found to be the most IgE-reactive [76]. 

For routine diagnostics, currently only a whole fenugreek extract is available as ImmunoCAP. There are no approved solutions for skin testing, i.e., only the prick-to-prick test with suspended fenugreek seed flour can be used to detect a sensitization on the skin. 

The cross-reactivity between fenugreek and other legumes is difficult to assess due to few published cases. In 2009, a study was published on a total of 31 patients, 29 of whom had positive IgE to peanut [72]. In this work, an oral challenge dose of 2 mg of natural fenugreek powder gave rise to the occurrence of objective allergic symptoms. Sensitization to fenugreek was considered to be secondary to existing peanut sensitization. Only one primary sensitization to fenugreek was found. A relatively recent case report from Germany also describes fenugreek anaphylaxis in a 34-year-old female peanut-allergic patient [77]. However, fenugreek anaphylaxis has also been described in a child with lentil and fava bean allergy [78]. A very recent study investigated the extent of cross-reactivity among legumes in 195 peanut-allergic children; the results showed sensitizations to lupine, fenugreek, soybean, and lentil in decending order of frequency [66]. 

Despite the potential cross-reactivity with other legume allergen sources, especially peanut, there is no mandatory declaration so far, which is certainly due to the rare reports of fenugreek anaphylaxis. 

### 4. Evaluation of nuts and seeds with regard to allergies 

Nuts and seeds are important triggers of food-induced anaphylaxis [79, 80] and a frequent cause of severe to fatal reactions [51, 81]. They contain a variety of potent allergens (Table 7). On the other hand, they represent a nutritional mainstay of the vegan diet. 

Recent data from the UK show an increase in the incidence of food-induced anaphylaxis [51], and data from the USA show the most significant increase for anaphylaxis triggered by nuts and seeds [82]. A Swedish study showed that reactions to cashews in particular have increased [83]. In the German anaphylaxis registry, triggers differ between children and adults [39]. Among nuts, reactions to cashew, hazelnut, and walnut have been reported most frequently (Figure 1), although no report of cashew-induced anaphylaxis in adults has been reported for German-speaking countries to date. The major challenge in diagnostics is the classification of clinically relevant cross-reactions among nuts. To date, the best described and most common clinically relevant cross-reactions are between cashew and pistachio and between walnut and pecan [84, 85, 86, 87]. 

**4.1. Cashew**

Cashews are kernels (called nuts) of the cashew apple and part of the *Anacardiaceae* family. 

They are among the most widely produced nuts in the world, and consumption is increasing. For example, cashew nut imports to Finland increased by a factor of 37 from 2002 to 2019, and the proportion of cashew nut-induced anaphylaxis increased in parallel [88]. Cashews are primarily consumed in roasted form, but are also used as an ingredient in many processed foods such as pesto, pastries, and confectionary. 

In the allergen database of the WHO/IUIS Allergen Nomenclature Subcommittee, three storage proteins of cashew nut are registered, the vicilin-like protein Ana o 1, the legumin-like protein Ana o 2, and the 2S albumin Ana o 3 (http://www.allergen.org). In a German study, sensitization to Ana o 3 was highly predictive of the presence of clinically relevant cashew allergy in children at 2.0 kU/L [89]. In addition, Bet v 1-homologous PR-10 protein isoforms were identified [90]. The exact prevalence of cashew nut allergy is not known. In older studies, 30% of nut-allergic individuals in the USA and 20% in the Netherlands were allergic to cashews (summarized by Borres et al. 2022 [91]). Cashew was also the most common trigger of nut-induced anaphylaxis in Europe [50]. 

1 mg of cashew protein, corresponding to one hundredth of a kernel, caused an objective allergic reaction in 11% of cashew-allergic children. Thus, even significantly smaller amounts than described for peanut allergy can lead to severe allergic reactions [92]. However, German data could not confirm this [49]. 

The most common cross-reactions are to pistachios, which also belong to the *Anacaridaceae* family. In the so-called “Nut-cracker” study, all patients with pistachio allergy were also allergic to cashew, and 65% of cashew-allergic patients developed pistachio allergy [85]. In a follow-up study, the same research group demonstrated that oral immunotherapy with cashew also resulted in tolerance of all co-sensitized pistachio-allergic patients [93]. Other cross-reactions have been described to allergens from within citrus seeds (summarized by Borres et al. 2022 [91]), although these must be released first by destruction of the seeds to become clinically relevant. In addition, all patients with allergy to pectin described in the literature were also allergic to cashew seed, although the cross-reactive allergen has not yet been identified. 

**4.2 Hazelnut**

Hazelnut (*Corylus avellana*) belongs to the birch family (*Betulaceae*). A wide range of symptoms has been described for hazelnut in particular, from mild oral allergy symptoms to severe anaphylactic reactions. European data show that hazelnut allergy is one of the most common food allergies in Europe [94, 95]. In adults, it is the most common sensitizing nut. However, there are large geographical differences in this regard [96]. In Central Europe, hazelnut allergy, when studied in the general population, is mostly pollen associated and gives rise to mild symptoms [97]. Nevertheless, data from the anaphylaxis registry (Figure 1) show that hazelnut is one of the most common triggers of food-induced anaphylaxis. The frequency of hazelnut allergy confirmed by oral food challenge is 1% in children [98]. Results of a study with participating clinics from London, Geneva, and Valencia showed that 32% of “nut”-allergic children had an allergy to hazelnut by oral food challenge [85]. 

The high prevalence of hazelnut allergy in individuals with allergies to other nuts and seeds can be explained by the high homology between the allergenic structures (Table 7): On the one hand, PR-10 proteins play a role and are responsible for the widespread cross-sensitization to several PR-10 proteins of various fruits, seeds, and nuts; on the other hand, storage proteins such as Cor a 14 (2S albumin), Cor a 9 (11S globulin), and Cor a 11 (7S vicilin) are relevant and have been associated with severe allergic reactions [99, 100]. 

**4.3 Sesame**

Sesame (*Sesamum indicum*) has been cultivated since 3500 BC, mainly in India and Africa, and is the oldest known edible seed. Sesame seeds are mainly used to make sesame oil and tahini. Tahini is a paste made from peeled, roasted, and finely ground sesame seeds, which is used primarily in halva, hummus, but increasingly also in various dips and sauces. Whole sesame seeds are also used as a topping for pastries, breads, etc., but there are studies that question the allergenic relevance of intact seeds, possibly explaining discrepancies in the medical history of some patients [101]. The wide use of sesame seeds in the food industry poses a risk for unintentional ingestion of “hidden” sesame [102]. To better protect allergic consumers, sesame is included in Annex II of the LMIV as one of the allergens subject to declaration in the EU. 

Sesame is a common cause of food allergy and anaphylaxis. In a questionnaire-based cross-sectional study in the USA, the prevalence of sesame allergy was 0.23%, with 24% reporting a severe sesame-induced allergic reaction [103]. Disturbingly, given the prevalence of severe reactions, the diagnosis of sesame allergy is difficult, particularly in adults. In a British study, of 10 patients with positive oral challenge to sesame, some of which had severe allergic reactions, 90% had no detectable IgE to sesame in vitro or negative skin tests with extracts or various sesame products [104]. 

Seven sesame allergens from four allergen families are registered in the allergen database of the WHO/IUIS Allergen Nomenclature Subcommittee: the 2S albumins Ses i 1 and Ses i 2, the vicilin-like protein Ses i 3, the oleosins Ses i 4 and Ses i 5, the legumin-like allergens Ses i 6 and Ses i 7 (http://www.allergen.org). For routine diagnostics, only Ses i 1 is currently available. Based on an analysis of 246 positive oral challenges with sesame, it was calculated that 5% of sesame-allergic patients react to a protein amount of 2.4 mg and 10% react to 7 mg. Based on this analysis, 4 g of tahini paste, corresponding to one teaspoon, cause objective allergic symptoms in 93% of sesame-allergic patients [105]. Baked intact sesame seeds may be less allergenic and should be evaluated separately from sesame paste [101]. 

**4.4. Hemp**

Hemp seeds – also known as hemp nuts – are nowadays used more and more due to their nutritionally favorable composition (https://hashmuseum.com/de/cannabis-wissen/hanf-als-nahrungsmittel/). Hemp oil contains more than 90% polyunsaturated fatty acids. However, these are mainly alpha-linolenic acid, which can only be converted to the physiologically required long-chain omega-3 fatty acids EPA and DHA to a very limited extent. 

IgE-mediated reactions to hemp are rare [106]. Symptoms of cannabis allergy can be expressed either via the respiratory tract (respiratory allergy) or as contact urticaria on the skin following direct contact, for example during harvesting or production. 

To date, several allergens of *Cannabis sativa* have been described. Depending on the cohort, one of the most important allergens is Can s 3, an nsLTP, which can cause reactions either primarily or in the context of cross-reactivity to other LTPs, for example peach or mugwort [107]. In patients with specific IgE reactivity to nsLTP due to pollen-associated and/or vegetable-associated food allergy, sensitization to *C. sativa* can be observed in up to 25% [108]. Another cannabis allergen that has been identified is a thaumatin-like protein (PR-5) with a molecular weight of 38 kDa and homologous to thaumatins in kiwi (Act d 2), apple (Mal d 2), and apricot (Q af 2), for example [109]. A profilin (Can s 2) has also been described [110]. A Bet v 1 homolog, Can s 5, has been documented by the WHO/IUIS (Table 7). 

Patients suffering from IgE-mediated cannabis allergy due to nsLPT sensitization may develop a so-called cannabis-fruit-vegetable syndrome [111]. Here, allergic reactions occur to various foods, such as peach, apple, nuts, tomato, and in rare cases to oranges and grapefruit. Not infrequently, reactions can be observed only in the context of cofactors, such as the concomitant use of NSAIDs. Other plant allergens that may cross-react with Can s 3 include allergens in latex as well as grapes and hops – the latter may also lead to intolerance of alcoholic beverages, such as wine and beer. Thaumatin-like proteins, which belong to the PR-5 family, can also lead to pronounced cross-reactivities between cannabis and plant foods (see above). 

### 5. Evaluation of wheat with regard to allergies 

The diverse clinical presentation of wheat-associated conditions poses a particular challenge [112]. Allergic reactions to wheat comprise a heterogeneous group of diseases that can be divided into different entities according to the triggering allergen structure (Table 8), pathomechanism, and clinical symptoms [22]. The spectrum of IgE-mediated reactions to wheat allergens ranges from baker’s asthma caused by inhaled allergens [113], primary wheat allergy occurring predominantly in childhood [114, 115], to wheat-dependent exercise-induced anaphylaxis (WDEIA) which is the most common form of food-dependent summation anaphylaxis [115, 116, 117, 118, 119]. 

IgE-mediated reactions following the consumption of wheat-containing products must be distinguished from celiac disease. In the latter, given a genetic predisposition, components of the gluten fraction of cereals cause villous atrophy via a T-cell-mediated immune reaction with subsequent functional damage to the small intestinal mucosa [120]. 

Wheat (*Triticum aestivum*) represents a staple food in the German and Central European diet and, due to its versatile food technological properties, finds widespread use in the food industry: in cookies, cakes, and cereals, in many sweets, pasta as well as in almost all conventional types of bread and rolls, wheat is found as the main ingredient. 

In the database of the WHO/IUIS Allergen Nomenclature Subcommittee, 28 allergens are listed, with more than 70 additional allergen structures already described. In addition to profilins, Tri a 14 (LTP) and Tri a 19 (omega-5 gliadin) are worth mentioning as clinically relevant allergen structures. Important for diagnostics – but also for processing – are the solubility characteristics (Osborne fractions). They are subdivided into water-soluble albumins, salt-soluble globulins, ethanol-soluble prolamins, and insoluble glutelins, the content of which varies depending on the type of grain. While the soluble protein fractions (albumin and globulin) are found in the aleurone layer and in the germ, the insoluble proteins of the gluten fraction (gliadin and glutenin) are contained in the endosperm, and on combining flour with water develop important binding properties required for baking (Table 8) [121, 122]. 

Although wheat is a frequent cause in infants and young children, anaphylactic reactions in childhood are quite rare [22]. One diagnostic problem is that wheat-specific IgE differentiates particularly poorly between clinically relevant sensitization and tolerant individuals. It must be kept in mind for any food allergy that sensitization is not synonymous with clinically relevant allergy. However, wheat sensitization in particular is frequently observed but rarely clinically relevant. In keeping with this, in a study in 106 children, wheat-specific IgE was also detected in 63% of those tolerant to wheat [114]. In addition, the spectrum of recognized wheat proteins was the same for allergic and tolerant children. The widespread opinion that older varieties are better tolerated in wheat allergy, due to a different protein profile, could not be confirmed [123]. Thus, spelt, khorasan wheat, emmer, einkorn, or triticale as hybrid strains of wheat must also be avoided in wheat allergy. In contrast, wheat is one of the more common causes of anaphylaxis in adults [22]. This is due to cofactor-dependent wheat allergy, which requires augmentation factors or increased levels of gluten consumption to be elicited [116, 118]. [Table Table9]

On the commercial market, wheat is traded in various states of processing. However, this has no influence on the allergenicity of the wheat allergen, only on the absolute content in the end product. For example, wheat products with a low degree of milling (lower type designation, for example type 405) have a higher gluten content compared to whole-grain products [124]. 

### 6. Increase in ultra-processed foods viewed critically 

A diet of freshly prepared and minimally processed foods requires both time and skills in terms of meal planning and preparation. It is best accomplished through good cooking skills, but generally requires more time for planning, purchasing, and preparation. This may tempt people to use more and more convenience products, including vegan foods, as a recent study shows [125]: unlike in Austria or Switzerland, the market for vegan and vegetarian substitute products in Germany shows a logarithmic increase. Suppliers of such products now offer a wide range of supposedly equivalent vegan alternative products that resemble the deliberately avoided animal-based foods in appearance, possible use, and taste. 

**Figure 3:**
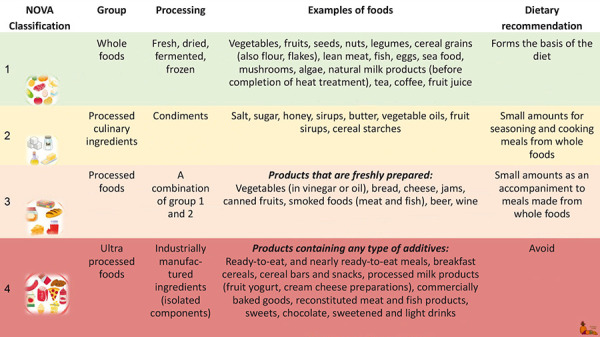
Classification of foods according to NOVA classification. Based on [128, 129].

According to the NOVA criteria defined by Monteiro (NOVA is not an acronym) (Figure 3) [126, 127], the majority of vegan alternative products are processed and ultra-processed foods (UPFs). 

UPFs differ from other foods by their ingredients (see below), but also by the reasons behind their production: they are intended to be “convenient” (nonperishable and ready to eat), tasty (with the goal of increasing consumption), and profitable (inexpensive ingredients, but added value when sold) [128]. These goals are achieved through the almost exclusive use of industrially pre-prepared ingredients. These include fractionated components such as proteins, fibers, starches, sugars, isolated carbohydrate compounds, and refined oils and fats that are chemically modified. The (ultra-processed) ingredients are extruded, molded, or pre-cooked (e.g., roasted or fried) and subsequently combined with other components [126, 127]. The result of this processing are products that are characterized by high energy but low nutrient density. Typically, ready-to-eat UPFs contain high levels of salt, sugar, and starch, but low levels of protein, fiber, and micronutrients. Thus, they closely resemble the dietary pattern that has been blamed for adverse health effects [9, 128]. Therefore, critical evaluation of vegan substitute products is essential.

**BOX 7: **Dietary patterns that favor ready-to-eat ultra-processed foods are high in salt, sugar, and starch but low in protein, fiber, and micronutrients.

The production of meat substitutes increased by 37% to 20,000 tons within 1 year in Germany alone [129]. According to a recent study from France, 31.1% of the population’s daily food selection consists of UPFs [130]. In particular, people in the age group between 18 and 39 years living in an urban environment are receptive to these products according to this study. Due to the growing market for UPFs in the field of vegan substitute products, it can be assumed that these results can be transferred to other countries of Western civilization. 

Should the proportion of UPFs in the diet of vegans increase, then the nutritional benefits of a plant-based diet as observed in its traditional form can be expected to disappear in the long run. The aim to consume UPFs in order to provide fortified nutrients should also be questioned, as the macro- and micronutrient deficiencies of vegan diets can only partially be compensated by consumption of UPFs (Table 3). 

A comparison between traditional diets that are reduced, low in, or free from animal products (flexitarian, vegetarian, and vegan) and contain traditional vegan products (e.g., tofu or hummus) with modern diets that use plant-based but highly processed “alternative” foods in exchange for meat, eggs, and dairy products impressively shows that the switch is associated with a clearly less favorable nutrient profile [16]. On the one hand, there is an increase in the intake of saturated fatty acids, sodium and, to some extent, sugar. On the other hand, a decrease in the intake of calcium, potassium, magnesium, zinc, and vitamin B12 can be observed. In the vegan diet, the change from traditional to modern is accompanied by a reduction in fiber intake of more than half. Although looking at individual ingredients to assess the quality of the overall diet is generally not useful [131], conclusions can be drawn based on the changed nutrient profile of this comparative study. For example, the altered potassium-sodium ratio is a clear indicator of a lower proportion of vegetables and low-processed foods. Similarly, the dramatic decrease in fiber intake in the vegan diet indicates the use of highly processed foods. Thus, the diets of all three groups (flexitarian, vegetarian, and vegan) using vegan UPF alternative products differ little from the undesirable Western (omnivorous) diet (“Western diet”) with all its health risks [9]. 

However, the increasing use of highly processed foods is not only problematic from a nutritional and health perspective. With regard to allergies and from gastroenterological and immunological view points, it can be assumed that the lack of fermentable dietary fiber, secondary plant metabolites, and other valuable natural ingredients could also have a negative effect on the microbiome, intestinal barrier, and have unfavorable consequences on the immune response [9, 132, 133]. To prevent allergic diseases, a varied diet is recommended, which should consist of meals prepared as freshly as possible [36, 134, 135]. Animal-based foods such as yogurt, raw milk, eggs, and fish have been found to be particularly protective [136, 137, 138].

**BOX 8: **From an allergological, gastroenterological, and immunological point of view, dietary patterns characterized by high food diversity and minimally processed foods are preferable.

### 7. Research needs and demands 

Research needs:

- To determine the stability and amount of storage proteins and PR-10 homologs in selected protein products (isolates, concentrates)
- To analyze allergenic activity of (highly) processed foods
- To examine processing forms with regard to clinical relevance for allergy sufferers

Demands:

- More precise labeling of proteins/concentrates and isolates with regard to their protein content
- Review list of foods subject to allergen labeling and expand if necessary
- The disclosure of unintended allergen presence due to cross-contact/ cross-contamination should be regulated by law
- The “vegan” label should not only be based on the list of ingredients, but should also include unintended cross-contact/cross-contamination with animal-based components and should ideally be legally regulated

### 8. Conclusion 

As plant foods are the major food allergens with increasing age, the current trend towards a long-term vegan diet must be viewed critically. At the same time, the allergenic potential of many highly processed products on the market cannot be fully assessed due to various gaps in research. In terms of allergy prevention, a vegan diet not only contradicts the recommendation for a varied diet consisting of as many food groups as possible, it also lacks particularly the foods associated with protective effects regarding allergic diseases. 

From a nutritional point of view, vegan means that many nutrients are missing or not available in sufficient quantities. To ensure an adequate supply of vitamin B12, but also of calcium, iron, iodine, zinc, as well as high-quality protein and long-chain omega-3 fatty acids (EPA/DHA), an in-depth study of the subject of nutrition, time investment, and supplementation, with regard to various nutrients, is required. Fortification of foods is quite rare in Germany and is only permitted in exceptional cases for organically produced foods. An European Court of Justice‘s ruling in 2021 has led to calcium-enriched “milk substitute” products now being the exception rather than the rule. This results in the often already poor calcium supply becoming even more critical when switching from milk to so-called “milk substitute” products. 

Expert nutritional counseling is recommended for those following or those with a tendency to follow vegan diets. A vegan diet is particularly critical in infants and toddlers, as the required quantities needed to ensure adequate supply often exceed the capacity of (young) children. 

## Funding 

None. 

## Conflict of interest 

R. Treudler, S. Schnadt, S. van Dullemen, B. Ballmer-Weber, S. Müller, U. Jappe declare no conflict of interest. 

I. Reese declares the following conflicts of interest outside the submitted work: Honoraria: AGPAS e. V., Aimmune Therapeutics Germany GmbH, AlbertZwei media GmbH, ALK-Abello Arzneimittel GmbH, Beiersdorf Dermo Medical GmbH, BVDD e. V., DAAB e.V., DAEM e.V., Danone Deutschland GmbH, DAPM e. V., DDG e.V., DGAKI, DWA, GPA e.V., GEKA mbH, GWT-TUD GmbH, Helmholtz-Zentrum München, InfectoPharm Arzneimittel und Consilium GmbH, Kneipp-Ärztebund e.V., Leo Pharma Gmbh, Medical Project Design, MVS Medizinverlage, Nestlé Deutschland AG, Netz für entzündliche Dermatosen in Hannover e.V., Novartis Pharma GmbH, RG Ärztefortbildung, Sanomega GmbH, Schweizer Milchproduzenten SMP, SWR, Uniklinikum Augsburg, Uniklinikum TU München, Uniklinikum Marburg, VDD e.V., VDOe e.V., Vfed e.V. Publishers: Dustri, de Gruyter, Springer, Thieme, Münchner Verlagsgruppe. Reimbursement of travel costs: DGAKI, EAACI, GA2LEN GAFA. Advisory board activity: DAAB, DGAKI. 

C. Schäfer declares the following conflicts of interest outside the submitted work: Travel costs, honoraria: abbvie Deutschland GmbH & Co. KG, Aimmune Therapeutics Germany GmbH, DAAB e. V., DAEM e. V., DGAKI e. V., DGEM e. V., Dustri Verlag, GU Verlag, MVS Medizinverlage, Nestle Deutschland AG, Novartis Pharma GmbH, Roxall Medizin GmbH, Sanofi-Aventis Deutschland GmbH, Springer Verlag, Thieme Verlag, VFED e. V. Advisory board activity: DAAB e. V., NNI, Quetheb e.V. 

K. Beyer declares the receipt of consultation fees and/ or honoraria by Danone/Nutricia, Hipp und Nestlé, as well as research grants by Danone/Nutricia and Hipp outside the submitted work. 

M. Worm declares the receipt of honoraria and/or consultation fees by the following companies: Novartis Pharma GmbH, Sanofi-Aventis Deutschland GmbH, DBV Technologies S.A, Aimmune Therapeutics UK Limited, Regeneron Pharmaceuticals, Inc, Leo Pharma GmbH, Boehringer Ingelheim Pharma GmbH &Co.KG, ALK-Abelló Arzneimittel GmbH, Lilly Deutschland GmbH, Kymab Limited, Amgen GmbH, Abbvie Deutschland GmbH & Co. KG, Pfizer Pharma GmbH, Mylan Germany GmbH (A Viatris Company), AstraZeneca GmbH, Lilly Deutschland GmbH and GlaxoSmithKline GmbH & Co. KG. 

S. Dölle-Bierke declares the receipt of honoraria by ALK and DAAB e. V., as well as research grants by BMBF (01EA2107B) outside the submitted work 

**Table 1.** Daily vegan eating plan covering requirements for an adult with a body weight of 60 kg (German Federal Food Code) [12].

**Meal**	**Ingredients**	**Quantity ** **(g)**	**Energy ** **(kcal)**	**Protein ** **(g)**	**Lysine ** **(mg)**	**Cysteine/Methionine (mg)**
**1** ^st^ ** breakfast**	**Banana porridge**					
	Oats whole-grain flakes	70	248	9.3	320	402
	Soy drink + calcium	200	54	6.9	440	100
	Banana	125	113	1.4	71	14
**2** ^nd^ ** breakfast**	**Whole-grain bread with peanut/chocolate spread and raw vegetables**					
	Whole-grain rye bread	70	139	5.1	224	168
	Peanut butter	25	145	6.6	258	165
	Cocoa low in oil (“baking cocoa”)	4	14	0.9	29	22
	Carrots, raw	150	50	1.3	71	32
**Lunch**	**Indian potato and lentil stew**					
	Potatoes, peeled cooked	200	140	3.8	248	106
	Lentils, ripe dried cooked	150	183	15.8	1127	281
	Tomatoes	150	26	1.4	54	16
	Onion, cooked	30	9	0.4	21	16
	Rapeseed oil	10	106			
	Iodized fluoridated table salt	2				
**Snack**	**Strawberry soy yogurt with walnuts**					
	Soy yogurt with calcium	200	54	6.9	442	100
	Strawberries (raw)	125	40	1.0	43	10
	Walnuts	30	214	4.8	133	155
**Dinner**	**Pan-fried vegetables with tofu**					
	Tofu	80	106	13.1	817	343
	Chickpeas	120	169	9.0	637	216
	Mixed vegetables, cooked	250	98	6.8	505	218
	Rapeseed oil	12	106			
	Sesame, roasted	10	60	1.9	59	83
	Iodized fluoridated table salt	2				
**Total**			**2,074**	**96.4**	**5,499**	**2,447**

Note: Daily requirement of lysine is 4.61 g, of cysteine and methionine 2.21 g. Macronutrient profile: 18.1 energy (E)% protein, 32.48 E% fat, 42.98 E% carbohydrates [13].

**Table 2.** DIAAS of selected food proteins (partly isolated) according to [20].

**Food**	**DIAAS ** **0.5 – 3 years**	**DIAAS ** **> 3 years**	**Protein content %**
Potato protein	96 – 113	120 – 134	78 – 81% (protein content potato: 2%)
Wheat	34 – 64	43 – 76	11 – 16
Oatmeal	48 – 62	57 – 73	10 – 15
Fava beans	49 – 59	57 – 69	25 – 27
Pea	54 – 77	69 – 91	18 – 28
Lupine	63 – 69	74 – 81	31 – 36
Soybean, dried	82 – 97	8 – 125	35 – 37
Soy protein isolate	75 – 84	88 – 98	85 – 93
Soy protein concentrate	86 – 97	101 – 112	64 – 65
Ham, smoked	115 – 125	123 – 142	24 – 34
Whey powder	81 – 87	101 – 109	12 – 13 (whey content in milk: 0.6 – 1%)
Casein	113 – 120	132 – 141	87 – 89 (casein content in milk: 2.6%)

DIAAS = digestible indispensable amino acid score.

**Table 3.** Combination ratios for a meal with ~ 20 g of protein providing a complete protein in terms of the digestible indispensable amino acid score (DIAAS); according to [12, 21].

	**Protein sources in g food**	**Percentage of proteins**	**Total protein content (g)**
Protein 1	73 g pea (cooked)	25	20.0
Protein 2	44 g wheat (whole grain/cooked)	25	
Protein 3	530 g potato	50	
Protein 1	15 g oats (whole grain)	10	20.0
Protein 2	43 g lentils (cooked)	20	
Protein 3	740 g potato	70	
Protein 1	33 g soybeans (cooked)	25	20.0
Protein 2	97 g wheat (whole grain/cooked)	20	
Protein 3	580 g potato	55	
Protein 1	160 g corn (whole grain/cooked)	25	20.0
Protein 2	790 g potato	75	
Protein 1	146 g wheat (whole grain/cooked)	30	20.0
Protein 2	740 g potato	70	
Protein 1	64 g lentil (cooked)	30	20.0
Protein 2	740 g potato	70	

**Table 4.** A Comparison of the nutrients of cow’s milk with selected plant-based drinks (German Federal Food Code 3.01; Alnatura 2022; Alpro 2022; Bedda 2022; Harvest Moon 2022; Luve 2022; Oatly 2022; Provamel 2022; Rewe 2022).

**Products**	**Energy ** **(kcal/ 100 mL)**	**Fat ** **(%)**	**Carbohydrates ** **(%)**	**Protein ** **(%)**	**Calcium ** **(mg)**
**Milk alternatives**					
Cow’s milk (whole milk)	65	3.5	4.7	3.4	120
Soy drink (e.g., Alpro Sojadrink Bio (i.e. organic soy drink))	38	1.8	2.3	3.0	12.31
Soy drink (e.g., Alpro Sojadrink Original mit Calcium (i.e. plain soy drink with calcium))	39	1.8	2.5	3.0	120
Lupine drink (e.g., Luve aus Lupinen Natur (i.e. plain lupine drink))	50	1.5	7.2	1.0	120
Almond drink (e.g., Provamel organic-bio Mandel (i.e. plain organic almond drink))	38	2.5	2.4	1.0	–
Cashew drink (e.g., Provamel bio-organic Cashew No sugars (i.e. unsweetened organic cashew drink))	38	2.8	1.3	1.2	–
Coconut drink (e.g., organic-bio Barista Kokos (i.e. organic coconut drink with foaming properties))	36	1.5	4.7	0.4	–
Coconut drink (e.g., Alnatura Kokosdrink ungesüßt (i.e. unsweetened plain coconut drink))	12	0.5	1.3	1.7	–
Oat drink (e.g., Oatly Haferdrink Calcium (i.e. calcium fortified oat drink))	46	1.5	6.7	1.0	120
Oat drink (e.g., Oatly Haferdrink Barista Edition (i.e. oat drink with foaming properties))	59	3.0	6.6	1.0	120
Spelt drink (e.g., Alnatura Dinkeldrink ungesüßt (i.e. unsweetened spelt drink))	42	1.5	6.2	0.8	–
Rice drink (e.g. Provamel organic-bio Rice)	54	1.1	11.0	0.1	–
**Yogurt alternatives**					
Cow’s milk yogurt 3.5% fat	69	3.8	4.4	3.9	120
Alpro Natur Ohne Zucker (i.e. plain unsweetened soy yogurt alternative)	42	2.3	–	4.0	120
Alpro Heidelbeere (i.e. soy yogurt alternative with blueberry flavour)	70	0.4	8.2	3.7	120
Alpro Natur Mit Mandeln (i.e. plain soy yogurt alternative with almonds)	54	2.8	2.3	3.9	120
Harvest Moon Coconut Natur - Bio (i.e. organic plain coconut yogurt alternative)	128	11.9	4.5	0.8	–
Luve Lughurt Natur (Lupinenjoghurt – i.e. plain lupine yogurt alternative)	101	5.5	10.0	1.7	–
Luve Lughurt Mango (i.e. lupine yogurt alternative with mango flavour)	106	5.0	14.0	1.5	–
Rewe Bio & vegan Kokos Natur (i.e. plain organic coconut yogurt alternative)	97	7.7	5.1	1.4	–
Luve Lughurt Natur zuckerfrei (i.e. plain unsweetened lupine yogurt)	96	6.0	8.5	1.8	–
Vegan spreadable cream (e.g., Bedda Frischcrème natur)	300	30.5	1.0	4.0	–

Alnatura (Alnatura Produktions- und Handels GmbH website): https://www.alnatura.de/de-de/produkte/suche/ (05.08.2022); Alpro (Alpro GmbH), website: https://www.alpro.com/de/produkte/drinks/sojadrinks-natur/sojadrink-original-mit-calcium/ (05.08.2022); Bedda (Ethiconomy Services GmbH), website: https://bedda-world.com/produkte/ (05.08.2022); Harvest Moon (Whollees GmbH), website: https://harvestmoon.de/products#milk_alternatives (05.08.2022); Luve (Prolupin GmbH), website: https://madewithluve.de/produkte/ (05.08.2022); Oatly (Oatly AB, Malmö, Schweden), website: https://www.oatly.com/de-de/stuff-we-make (05.08.2022); Provamel (Alpro GmbH), website: https://www.provamel.com/de/produkte (05.08.2022); Rewe (Rewe Markt GmbH), website: https://www.rewe.de/marken/eigenmarken/bio/rewe-bio-produkte/ (05.08.2022).

**Table 5.** Comparison of cheese, cream cheese, and yogurt with alternatives (focus on protein) (Alpro 2022; Bedda 2022; Simply V 2022) [12].

**Product**	**Cheese from cow’s milk (reference) versus vegan alternative**	**Protein content**	**Calcium**	**Allergens**	**Trace identification**
Semi-hard cheese	Semi-hard cheese 45% FDM	25.3%	690 mg	Cow’s milk	
	Simply V® Vegane Genießerscheiben Fein Cremig (i.e. slices cheese alternative)	0.5%	–	Almonds	+
	Bedda® Scheiben Classic (i.e. slices cheese alternative)	0.0%	+	–	–
Cream cheese	Cream cheese heavy cream (60% FDM)	11.3%	79 mg	Cow’s milk	
	Simply V® Streichgenuss Cremig-Mild (i.e. cream cheese alternative)	4.5%	–	Almonds	+
	Bedda® Frischcreme Kräuter (i.e. cream cheese alternative with herbs)	4.0%	–	Almonds	–

FDM = fat in dry matter. edda® (Ethiconomy Services GmbH), website: https://bedda-world.com/produkte/ (05.08.2022); Simply V® (E.V.A. GmbH) website: https://www.simply-v.de/de/produkte/ (05.08.2022).

**Table 6.** Nutritional values and selected allergenic structures of selected legumes for estimation of possible risks for cross-reactive allergic reactions (based on 100 g ready-to-eat final product) according to Allergome (www.allergome.org); allergens that are additionally documented in the WHO/IUIS database (www.allergen.org) are presented in bold.

**Food**	**Energy (kilocalories) [fat in g, carbohydrates in g, protein in g]**	**Important allergenic structures**	**Notes (focus allergy/food form)**
Fenugreek *Trigonella foenum-graecum*	366 [6.4/48.3/23.0]	7S vicilin: Tri fg 1: 2S albumin: Tri fg 2 7S globulin: Tri fg 3 Bet v 1-like: Tri fg 4	No WHO/IUIS documentation yet Note: is increasingly used in usual spice quantities
Peanut *Arachis hypogaea*	599 [48.4/7.48/29.78]	7S vicilin: **Ara h 1** + isoforms 2S albumin: **Ara h 2/Ara h 6/Ara h 7** + isoforms 11S globulin: **Ara h 3 ** Profilin: **Ara h 5 ** Bet v 1-like: **Ara h 8** + isoforms LTP: **Ara h 9/Ara h 16/Ara h 17** + isoforms Oleosins: **Ara h10/Ara h 11, Ara h 14/Ara h 15** + isoforms	Peanut products are available in many variants (roasted, defatted, as flour, or as oil), resulting in significantly differing macronutrient contents.
Peanut roasted	630 [52.95/9.28/26.94]		
Peanut flour defatted flour	349 [0.55/30.65/52.50]		
Peas, ripe-flour *Pisum sativum*	316 [1.51/44.0/24.1]	7S vicilin: **Pis s 1** + isoforms 7S vicilin/Convicilin + isoform: **Pis s 2 ** LTP + isoform: **Pis s 3 ** Profilin: Pis s 5 Bet v 1-like: Pis s 6	Pis 1 and Pis 2 – together with isoforms probably the main allergens
Chickpea, dried *Cicer arietinum*	337 [5.9/44.0/19.0]	7S vicilin: **Cic a 1 ** 2S albumin: Cic a 2S ilbumin LTP: Cic a 3 11S globulin: Cic a 6	Chickpea flour contains 20% protein, twice as much as wheat flour
Lentil, dried ripe *Lens culinaris*	307 [(1.6/40.5/23.4]	7S vicilin: **Len c 1** + three isoforms LTP: **Len c 3** + isoform No 2S albumin or Bet v 1 homolog described so far	
Lupine, grist *Lupinus* species	356 [8.7/13.0/43.0]	7S vicilin: Lup a 1/**Lup** **an 1** major allergen, cross-reaction between peanut and lupine. LTP: **Lup an 3** + isoform Bet v 1-like: Lup a 4 Profilin: **Lup a 5** + isoform 2S albumin: Lup an delta conglutin	Based on the sequence homologies of lupine products, a similar allergenic potency for birch pollen allergen can be assumed for patients as with soybean
Soybean, dried *Glycine maxima*	386 [18.3/6.3/38.2]	Hydrophobic protein: **Gly m 1 ** Defensin: **Gly m 2 ** Profilin: **Gly m 3** + isoforms Bet v 1-like: **Gly m 4** + isoform 7S vicilin: **Gly m 5** + isoforms 11S globulin: **Gly m 6** + isoforms Seed biotinylated protein: **Gly m 7 ** 2S albumin: **Gly m 8**	Soy products are available in many variants (roasted, defatted, as flour, or as oil), resulting in significantly differing macronutrient contents.
Soybean, roasted	431 [24.0/6.1/50.0]		
Soy protein (TVP)	291 [1.5/7.0/50.0]		

Nutritional information is based on the German Federal Food Code 3.02 [12] and is part of the Prodi/Optidiet nutrition software. TVP = textured vegetable protein.

**Table 7.** Nutritional values and selected allergenic structures of relevant nuts for the estimation of possible risks for cross-reactive allergic reactions (related to 100 g of the ready-to-eat final product) according to Allergome (www.allergome.org); allergens that are additionally documented in the WHO/IUS database (www.allergen.org) are presented in bold.

**Food**	**Energy (kilocalories) [fat in g, carbohydrates in g, protein in g]**	**Important allergenic structures**	**Notes ** **(focus allergy/food form)**
Cashews *Anacardium occidentale*	590 [42.2/30.5/20.6]	7S globulin: **Ana o 1**, 11S gGlobulin: **Ana o 2 ** 2S albumin: **Ana o 3 (**f443, major allergen), Bet v 1-like isoform	
Cashew flour, de-oiled *Anacardium occidentale*	356 [2.0/41.8/39.6]		Basic ingredient for cashew processing (for example, cheese alternatives, drinks, and snacks).
Hazelnut, natural *Corylus avellana*	664 [63.3/6.0/16.3]	PR-10: **Cor a 1** and additional 10 isoforms Profilin: **Cor a 2** + 2 isoforms LTP: **Cor a 8** + isoform 11S globulin: **Cor a 9** + isoform 7S vicilin: **Cor a 11** + isoform 2S albumin: **Cor a 14** + isoform 7S globulin: **Cor a 16**	37 known allergen structures, of which 12 are documented at WHO/IUIS, but 2 are inhalant allergens. Various isoforms are known.
Hazelnut, roasted *Corylus avellana*	685 ([6.7/5.4/14.8]		

Nutritional information is based on the German Federal Food Code 3.02 [12] and is part of the Prodi/Optidiet nutrition software.

**Table 8.** Nutritional values and selected allergenic structures of relevant oilseeds and seeds for the estimation of possible risks for cross-reactive allergic reactions (related to 100 g of the ready-to-eat final product) according to Allergome (www.allergome.org); allergens that are additionally documented in the WHO/IUS database (www.allergen.org) are presented in bold.

**Food**	**Energy (kilocalories) [fat in g, carbohydrates in g, protein in g]**	**Important allergenic structures**	**Notes (focus allergy/food form)**
Psyllium *Plantago ovata*	271 [7.0/3.5/16.0]	Pla o without further characterization	Note the significantly different nutrient profile compared to psyllium husks.
Psyllium husks *Plantago ovata*	222 [0.7/12.0/2.3]		
Hemp seeds, shelled *Cannabis sativa*	611 [48.0/16.0/25.0]	Profilin: **Can s 2** (identified as an aeroallergen) LTP: **Can s 3** + isoform (identified as an aeroallergen, not a food allergen). **Can s 4**: Oxygen Evolving Enhancer Protein 2 (identified as an aeroallergen). **Can s 5**: Bet v 1-like (identified as an aeroallergen)	Hemp seeds should preferably be used shelled. Unprocessed and unshelled hemp seeds must be pre-processed by heat to avoid the antinutritive substances.
Hemp seeds, unshelled *Cannabis sativa*	461 [32.0/2.2/21.0]		
Hemp seeds, roasted *Cannabis sativa*	560 [44.0/12.0/33.0]	Edestin (11S globulin), but not described as allergen!	
Linseed, whole *Linum usitatissimum*	498 [37.0/7.7/22.0]	2S albumin: Lin u 1	Observe EU Regulation on consumption restriction
Sesame, raw *Sesamum indicum*; Synonym (old): *Sesamum orientale*	593 [50.4/10.2/50.4]	2S albumins: **Ses i 1** + isoform, **Ses i 2** + isoform, 7S vicilin: **Ses i 3** + Isoform Oleosins: **Ses i 4**, **Ses i 5** + isoforms 11S globulin: **Ses i 6** + isoform 11S globulin: **Ses i 7** + isoform	

Nutritional information is based on the German Federal Food Code 3.02 [12] and is part of the Prodi/Optidiet nutrition software.

**Table 9. Table9:** Nutritional values and selected allergenic structures of relevant wheat products for the assessment of possible risks for cross-reactive allergic reactions (based on 100 g of the ready-to-eat final product) according to Allergome (www.allergome.org); allergens that are additionally documented in the WHO/IUS database (www.allergen.org) are presented in bold.

**Food**	**Energy (kilocalories) [fat in g, carbohydrates in g, protein in g]**	**Important allergenic structures**	**Notes (focus allergy/food form)**
Wheat grain *Triticum aestivum*	301 [1.8/59.6/11.7]	Profilin: **Tri a 12** and isoforms LTP: **Tri a 14** + isoforms Omega 5 gliadin: **Tri a 19**	Over 100 described allergen structures (including amylase inhibitors, gliadins, glutenins, serine protease inhibitors, thiol reductases); 15/28 allergens already documented at WHO/IUIS as food allergens.
Gluten	395 [4.0/7.0/81.0]		
Seitan (*Triticum aestivum*)	115 [1.7/1.8/22.4]		

Nutritional information is based on the German Federal Food Code 3.02 [12] and is part of the Prodi/Optidiet nutrition software.
