# Corrigendum of the article Reese I, Schäfer C, Ballmer-Weber B, Beyer K, Dölle-Bierke S, van Dullemen S, Jappe U, Müller S, Schnadt S, Treudler R, Worm M. Vegan diets from an allergy point of view – Position paper of the DGAKI working group on food allergy. Allergol Select. 2023; 7: 57-83

**DOI:** 10.5414/ALX02400ECorr

**Published:** 2024-06-13

Regarding the article by *Reese I, Schäfer C, Ballmer-Weber B, Beyer K, Dölle-Bierke S, van Dullemen S, Jappe U, Müller S, Schnadt S, Treudler R, Worm M.* Vegan diets from an allergy point of view – Position paper of the DGAKI working group on food allergy. Allergol Select. 2023; *7:* 57-83, the authors would like to apologize for an unfortunate error that occurred during translation of the German position paper into English: **not vitamin B12 but vitamin B2 was only met to a limited extent by the vegan group of the VeChi Diet Study** (page 60, left column, 3rd paragraph). Please find the correct version of the sentence below:

The results of the VeChi Diet Study (Vegetarian and Vegan Children Study), a German cross-sectional study of young children aged between 1 and 3 years with different diets (vegan, vegetarian, omnivorous) show that the requirements of critical micronutrients such as calcium and iodine as well as the long-chain omega-3 fatty acids are not met in vegans and those of vitamin B2 and iron (due to poorer availability from plant sources) are only met to a limited extent [17].

The error has been corrected in the HTML and PDF versions of the article.

*Imke Reese on behalf of the DGAKI working group on food allergy*

